# Evaluation of cervical posture improvement of children with cerebral palsy after physical therapy based on head movements and serious games

**DOI:** 10.1186/s12938-017-0364-5

**Published:** 2017-08-18

**Authors:** Miguel A. Velasco, Rafael Raya, Luca Muzzioli, Daniela Morelli, Abraham Otero, Marco Iosa, Febo Cincotti, Eduardo Rocon

**Affiliations:** 1 Neural and Cognitive Engineering Group, Centre for Automation and Robotics (CAR) CSIC-UPM, Ctra. Campo Real Km 0.2, 28500 Arganda del Rey, Spain; 2 Department of Information Technologies, Universidad CEU San Pablo, Urbanización Montepríncipe, 28668 Boadilla del Monte, Spain; 3 Fondazione Santa Lucia, FSL, Via Ardeatina, 306, 00142 Rome, Italy

**Keywords:** Cerebral palsy, Cervical posture, Inertial sensors, Serious games

## Abstract

**Background:**

This paper presents the preliminary results of a novel rehabilitation therapy for cervical and trunk control of children with cerebral palsy (CP) based on serious videogames and physical exercise.

**Materials:**

The therapy is based on the use of the ENLAZA Interface, a head mouse based on inertial technology that will be used to control a set of serious videogames with movements of the head.

**Methods:**

Ten users with CP participated in the study. Whereas the control group (n = 5) followed traditional therapies, the experimental group (n = 5) complemented these therapies with a series of ten sessions of gaming with ENLAZA to exercise cervical flexion–extensions, rotations and inclinations in a controlled, engaging environment.

**Results:**

The ten work sessions yielded improvements in head and trunk control that were higher in the experimental group for Visual Analogue Scale, Goal Attainment Scaling and Trunk Control Measurement Scale (TCMS). Significant differences (27% vs. 2% of percentage improvement) were found between the experimental and control groups for TCMS (p < 0.05). The kinematic assessment shows that there were some improvements in the active and the passive range of motion. However, no significant differences were found pre- and post-intervention.

**Conclusions:**

Physical therapy that combines serious games with traditional rehabilitation could allow children with CP to achieve larger function improvements in the trunk and cervical regions. However, given the limited scope of this trial (n = 10) additional studies are needed to corroborate this hypothesis.

## Background

Cerebral palsy (CP) is a disorder of posture and movement due to a defect or lesion in the immature brain [1]. It is the most common cause of permanent physical impairment in childhood and the prospect of survival in these individuals has increased in recent years [2]. CP affects between 2 and 3 per 1000 live-births [3], reported for the European registers by the Surveillance of Cerebral Palsy European Network (SCPE), which also presented a consensus on the definition [4], classification, and description of CP [5]. CP is often associated to sensory deficits, cognition impairments, communication and motor disabilities, behavior issues, seizure disorder, pain, and secondary musculoskeletal problems.

**Fig. 1:**
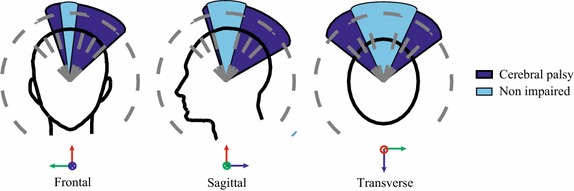
Angular orientations in frontal, sagittal and transverse planes measured in two people using a head mouse: an individual with hypotonic CP (*dark blue*) and a non-impaired user (*light blue*)

People with CP frequently show low performance in activities of the daily living (ADL) due to their limited limb, trunk, and head control. Unfortunately, the poor head and trunk control in CP produce limitations beyond function. An effective oral functioning for feeding begins with attaining better head stability to improve jaw control [6]. In individuals with cervical hypotonia (as shown in Fig. 1) the limitations can be so severe that the infant may experience difficulty swallowing and breathing.

### Assessment of cerebral palsy

When assessing people with CP, many factors and symptoms need to be monitored. The functional consequences of different health states are of special interest. The Gross Motor Function Classification System (GMFCS) has been widely employed internationally to group individuals with CP into one of five levels based on functional mobility or activity limitation [7]. So has the Manual Ability Classification System (MACS) [8], which describes manipulation skills. However, the assessment and conclusions in mild cases may vary by the subjective examinations of various professionals. Therefore, a combination of significant motor developmental delay and abnormalities in the neurologic examination is required to make a diagnosis. A promising approach is the use of normal and abnormal general movement patterns [9].

In the medical field there is a need for small ambulatory sensor systems for measuring the kinematics of body segments. Motion sensing, by means of inertial sensors, can provide a real breakthrough in this field [10]. As a result, inertial sensors have been chosen for different applications focused on people with CP. Examples of this are the evaluation of clinical spasticity [11], the quantification of standing balance by assessing displacement of the center of mass [12], and the assessment of the predominant motor signs [13].

### Therapies and treatments

The treatments for people with CP range from physical therapy to medication and surgery. Traditionally, they follow two basic principles [14]: emphasis on the normalization of the quality of movement; and emphasis on functional activities, which focuses on the development of skills necessary for the ADLs [15].

The priorities in the management of CP are currently changing. Whereas traditional strategies focused on promoting compensation, novel early treatments aim at restoring motor function. The reason behind this is the increasing knowledge about neuroplasticity, i.e. the ability of the neurons to reorganize their structure and function after an injury. The reorganization occurs in response to different factors, including physical training [16]. The task-oriented therapies aim to improve the movement and the posture of the patient by the repetitive training of a certain functional task. In children with CP, the research focuses on the assessment and treatment of the upper and lower extremities to improve their performance in ADLs. In contrast, the literature on the trunk and head control is scarce [17]. The physiotherapy for the promotion of postural control and balance consists of postural orientation exercises, and exercises to strengthen the neck, back, and the musculature of the upper limb. It also includes exercises to improve the relationship between the visual environment and an internal frame of reference [18]. This strategy can be very effective in the short-term. However, in long-term rehabilitation programs, the patient often loses motivation after repeating the very same exercises weekly. Once a patient starts losing focus on the program, the therapy loses effectiveness [19].

Some experiments such as those conducted by Kramer et al. [20], Lancioni et al. [21] and Shih et al. proved that the use of biofeedback training during several weeks yields improvements in head control which last up to 16 weeks once the training is discontinued. In those experiments, the biofeedback was provided by various systems: a head position trainer (HPT), a set of micro-switches, and the Nintendo Wii Balance Board [22].

### A new therapy based on videogames for physical rehabilitation

In this paper, we present a proof of concept of a rehabilitation therapy for the enhancement of head and trunk posture in children with CP. It is based on active head exercises performed through serious videogames controlled with ENLAZA, an interface based on inertial technology. We aim to develop evidence-based criteria for the integration of these exercises into the traditional therapies. Moreover, we intend to determine their role in maximizing head control in children with CP. We hypothesize that the users can improve their head posture by using the ENLAZA interface due to the neuroplasticity.

## Materials

### The ENLAZA interface

**Fig. 2:**
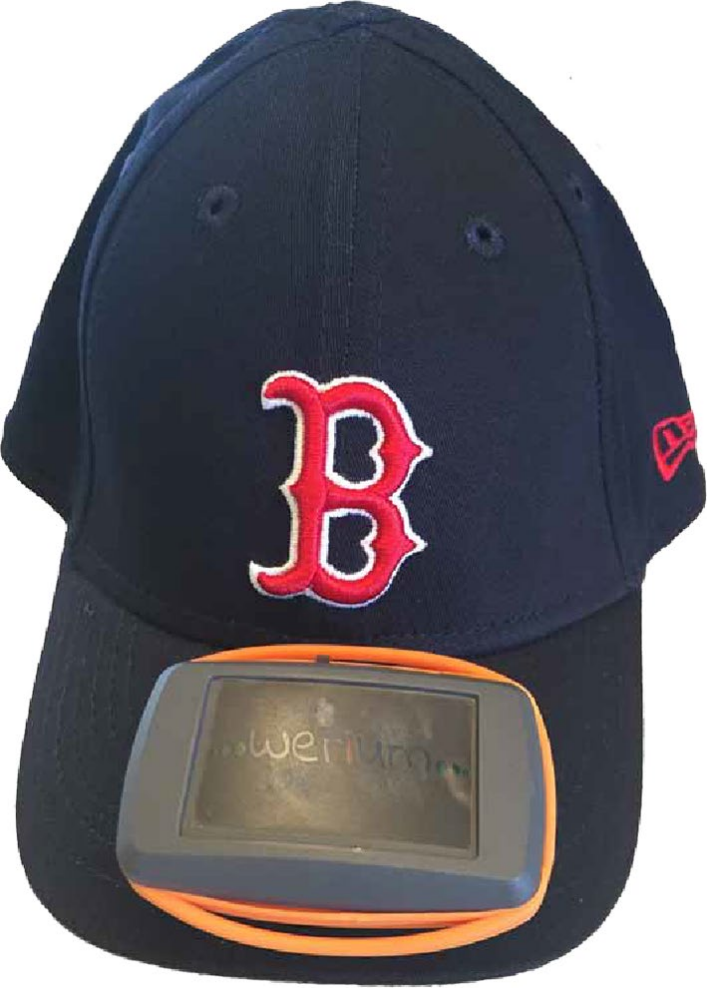
The ENLAZA interface (Werium Solutions S.L., Spain)

Raya et al. proposed the ENLAZA interface, an adapted input device for users with severe motor disorders (especially CP) who cannot use traditional solutions such as mice, joysticks or trackballs to access the computer [23]. The ENLAZA interface (depicted in Fig. 2) allows the users to control the cursor of the computer with movements of their heads. It consists of a head-set with a cap and an inertial measurement unit, IMU, (Werium S.L., Spain). ENLAZA integrates a tridimensional (3D) accelerometer, a 3D gyroscope, and a 3D magnetometer to measure 3D acceleration (caused by motion and gravity), 3D angular velocity, and 3D earth magnetic field. Its data fusion algorithm estimates the orientation of the IMU and enables the measurement of changes in inclination smaller than 1.0° and 1–2° of heading accuracy.

For the purpose of this study, the mouse pointer implements an absolute mapping, meaning that there is a one-to-one correspondence between head orientation and location of the pointer. After a calibration process, all pixels in the screen are reachable for the user’s cervical range of motion. During the calibration, a therapist adjusts the gain of the transfer function which translates the orientation of the head into a location of the pointer on the screen. A Robust Kalman Filter (RKF) was developed to facilitate fine motor control based on the characterization of involuntary movements found in users with CP [24].

### Serious games

The usefulness of the serious game relies on being designed to follow a series of requirements indicated by physiotherapists and considering the desirable features for rehabilitation [19, 25]:

- Meaningful play: The relationship between the player’s interactions and the responses of the system must be consistent.
- Engagement: The game must be challenging but not frustrating. By maintaining an optimal difficulty and by including motivational elements, the apparition of fatigue and boredom can be prevented.
- In addition, the inclusion of monitoring mechanisms simplifies the therapist’s work.

Six videogames were specially designed and developed in Visual C# and the framework .NET 4.0 to be played with the ENLAZA interface. additionally, another set of six commercial off-the-shelf videogames were adapted to be played with this system. These videogames have the characteristics enumerated above:

- They are fun and systematic: there are clear objectives for the user and the task and duration are detailed before the game starts.
- All the games have different levels of difficulty and they use colors or images to represent abstract concepts as time. In addition, the games provide the participants with visual and auditory feedback.
- There is a systematic record of all the scores achieved by the participants, the orientation of the head during the game and other parameters which measure task performance. Those metrics can be examined by the therapist for further analysis.

## Methods

**Table 1:** Inclusion/exclusion criteria for rehabilitation therapy based on head movements and serious videogames

Inclusion criteria	Exclusion criteria
Males and females, aged 4–21 years old	Aggressive or self-injure behavior
Diagnosed CP and cervical hypotonia or difficulties on head control	Involuntary movements of the head
Cognitive capacity and behavior appropriate to understand the tasks and follow simple instruction and active participation in the study	Cervical surgery within the previous 6 months
Signed written informed consent by parents or legal guardian	Inability to control the ENLAZA system during the first testing session
Medically stable	Severe visual limitations

Ten children with CP were recruited by the Fondazione Santa Lucia (FSL, Rome, Italy) to participate in this study. The inclusion/exclusion criteria can be found in Table 1. Five of the participants, the experimental group (aged 4.8 ± 3.0 years old), wore ENLAZA and played our selection of serious videogames. The five remaining children (aged 11.2 ± 3.8 years old) were grouped as controls for the experiment and followed the traditional physical and occupational therapy.

Randomization leaded to casual differences between the two groups at T0 (the beginning of the experiments). The impairment in the control group was on average more severe from a clinical point of view than that in experimental group, however these differences were not statistically significant (p = 0.222 for VAS, p = 0.421 for TCS, p = 0.095 for GMFM-88, Mann Whitney u test). A significant difference was found in terms of age (p < 0.05, t test), but it was higher in control group, so it should be considered as a potential disadvantage for experimental group. Furthermore, this difference should not be considered a critical issue because within-group analysis of the improvements will be calculated.

### Assessment of movement and posture

In the following sections, we present a kinematic and functional analysis of the improvement hypothesized. ENLAZA’s inertial sensor enabled the measurement of (1) the cervical range of motion (CROM) during active movements directly performed by the child and (2) the CROM during passive mobilization assisted by the therapist. The CROM consists of three movements: flexion–extension, rotation and lateral flexion.

Additionally, four measures were chosen to quantify the improvements in the posture of the head and trunk:

- Gross Motor Function Measure-88 (GMFM-88). The items 21 and 22 assess whether the children can lift and maintain their head in a vertical position with trunk support by a therapist while sitting [26].
- Visual Analogue Scale (VAS). It consists of a line of 100 mm separating two labels: 0 = “No head control” and 10 = “Perfect head control”. Parents, children, and therapists were asked to put a cross on the location of the line which they thought described best the children’s level of head control.
- Goal attainment scaling (GAS). It allows the therapist to program the desired improvement and to judge if the children achieved it. In this study, Goal 1 was related to head movement and Goal 2 was related to choking/swallowing.
- Trunk Control Measurement Scale (TCMS). It measures the children’s ability for static sitting balance, selective movement control, and dynamic reaching. It also gives insight into the strengths and weaknesses of the children’s trunk performance [17].

### Evaluation of task performance

The performance of the participants in the experimental group with during their game was quantified with two parameters: success rate and throughput.

- Success rate, *SR* (%), measures the number of successful movements performed to reach targets which moved vertically or horizontally.
- Throughput, *TP* (bits/s), is a well known parameter to quantify task performance during 1D and 2D pointing tasks. It can be calculated from the movement time (*MT*) and the index of difficulty (*ID*) of the task.
  1 $$\begin{aligned} {TP}_{avg} = \frac{ID_{avg}}{MT_{avg}} \end{aligned}$$
  The Shannon formulation used by [27] represents the *ID* as:
  2 $$\begin{aligned} ID = log_{2}\bigg (\frac{A}{W}+1\bigg ) \end{aligned}$$
  where *A* is the amplitude of the pointing movement and *W* is the width of the target.

### Procedure

At the beginning of a work session, the participants in the experimental group had to complete a routine to calibrate the interface. The procedure consisted in maintaining the head in front of the computer screen in a stable position for a few seconds. During the rest of the session, this head orientation located the cursor at the center of the screen during the game. The therapist would then set a value of the cursor-device gain which enabled the participant to reach all the pixels on the screen with head movements.

**Fig. 3:**
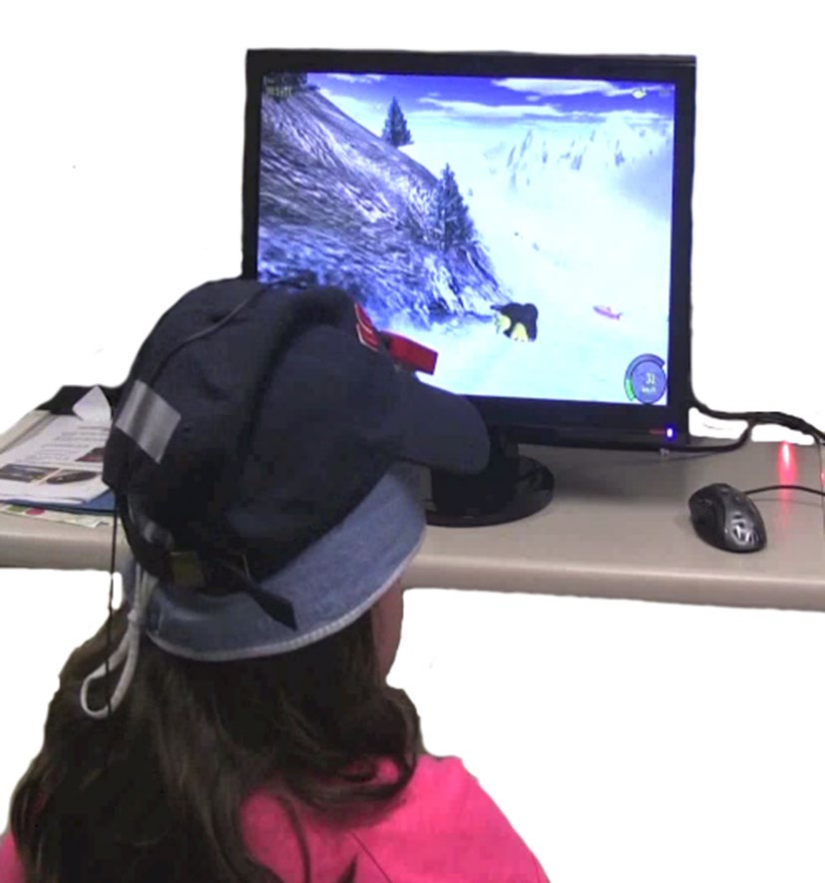
A training session with ENLAZA. The user has a wired IMU attached to a baseball cap and plays the game Extreme Tux Racer

The protocol in a work session of the experimental group was the following:

1. Measurement of the passive CROM.
2. Measurement of the active CROM.
3. Serious games controlled with ENLAZA.
  Practice time with free controlled off-the-shelf videogames.
  Reaching tasks with targets that moved vertically (falling targets) to exercise rotations.
  Reaching tasks with targets that moved horizontally to exercise the flexion–extension.
  2D pointing tasks. There were four series of four tasks of increasing difficulty, $ID_i$ = {1.32, 1.8, 2.0, 2.58 bits}. In this case, all the targets were static.

All the participants were sitting in front of a 17 in. computer screen, at a distance of 50 cm approximately as illustrated in Fig. 3. The screen resolution was 136 × 768 pixels. The whole session lasted about 25–30 min.

### Statistical analysis

The mean and standard deviation were used for the description of clinical scale scores. The percentage improvement was evaluated as

3 $$\begin{aligned} \textit{Improvement} = \frac{\textit{Post-value}-\textit{Pre-value}}{\textit{Pre-value}}\cdot 100 \end{aligned}$$

The non-parametric Wilcoxon signed ranks (WSR) test was used for within group analysis in order to compare clinical scores at T0 (the beginning of the experiments) and after the last work-session (T1), $\alpha$ = 0.05, whereas the Mann-Whitney u test was used for the between group comparisons at the T0 and T1, separately ($\alpha$ = 0.05).

## Results

### Functional assessment

**Table 2:** Clinical scores for experimental group (p value refers to Wilcoxon signed rank test)

Experimental group	Pre	Post	Sig.
VAS	6.4 ± 1.1	7.6 ± 1.3	$*\uparrow$
GAS	22.8 ± 0.4	64.3 ± 3.6	$*\uparrow$
TCMS	19.4 ± 47.5	24.2 ± 17.9	$*\uparrow$
GMFM-88	44.4 ± 0.20	50.2 ± 27.8	N.s.

Significance: * <0.05

Table 2 depicts the mean clinical scores pre- and post-intervention in the experimental group. The changes in the experimental group were statistically significant in terms of head control, visuomotor control assessed by GAS-score and TCMS (p < 0.05). The gross motor functioning improved slightly but the value of *p* did not achieve the statistical significant threshold. The items 21 and 22 of GMFM-88 remained unaltered in the experimental and control groups.

**Fig. 4:**
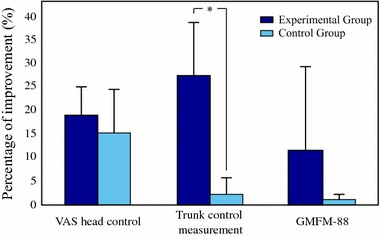
Mean and standard deviations of percentage improvements in the experimental group (*dark blue*) and the control group (*light blue*)

**Table 3:** Clinical scores for control group (p value refers to Wilcoxon signed rank test)

Control group	Pre	Post	Sig.
VAS	5.4 ± 1.1	6.2 ± 1.3	$*\uparrow$
GAS	24.8 ± 1.2	63.3 ± 5.9	$*\uparrow$
TCMS	9.6 ± 13.2	10.0 ± 13.7	N.s.
GMFM-88	23.0 ± 13.3	23.3 ± 13.6	N.s.

Significance: * <0.05

**Table 4:** Percentage improvements in clinical scores for experimental, vs. control group (p value refers to Mann Whitney u test)

Scale	Experimental	Control group	Sig.
VAS	18.9 ± 6.0%	15.2 ± 9.4%	N.s.
GAS	181.3 ± 17.0%	155.6 ± 27.9%	N.s.
TCMS	27.2 ± 11.5%	1.8 ± 4.1%	$*\downarrow$
GMFM-88	11.5 ± 18.7%	0.8 ± 1.77%	N.s.

Significance: * <0.05

In the control group (Table 3) statistically significant improvements occurred in terms of head control and visuomotor control (p < 0.05), but in terms of trunk control or gross motor functioning. It implied that the percentage of improvement of trunk control was significantly higher in the experimental group with respect to the control group (about +27% vs. +2%, respectively, as reported in Table 4). The other percentage changes, despite being higher in the experimental group, were not statistically different between the two groups, see Fig. 4.

### Kinematic assessment

The ranges of motion measured before and after the therapy showed a rise of active ROM in all three movements. The percentages of increment were +20, +38 and +85%, achieving 93°, 90° and 145° for active flexion–extension, lateral flexion and rotation, respectively. However the improvements were not statistically significant.

The passive ROM, presented smaller changes: an increment of +5 and +57%, achieving 77° and 140° of passive lateral flexion and rotation. Passive range of motion during flexion experienced a small reduction (−6%) and decremented from 86° to 81°. No statistical significance was found in these changes.

**Fig. 5:**
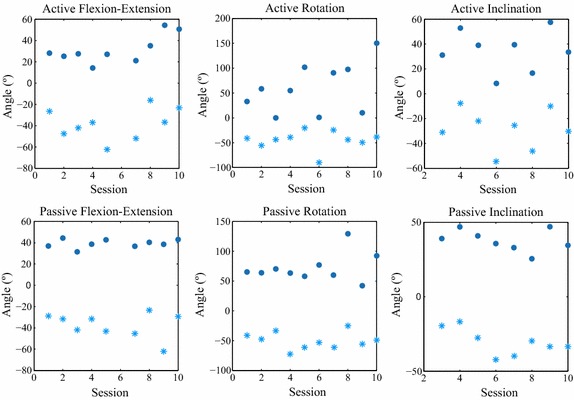
Evolution of the ROMs measured for the participant CP1.The* circle* represents the maximum angles measured; the* star* the minimum angles recorded

Figure 5 illustrates the evolution of active and passive flexion–extension, rotations, and inclinations of one of the participants in the experimental group.

### Task performance

**Fig. 6:**
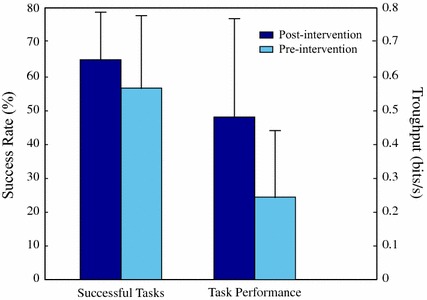
Mean values of task performance (success rate and throughput) pre- and post-intervention

The participants in the control group experienced an improvement in task performance after ten work sessions, but the differences were not significant. The mean value of success rate augmented from 56.8 to 65.1%, whereas the mean value of throughput also rose from 0.244 to 0.482 bits/s. These values are shown in Fig. 6.

## Discussion

This study aimed to quantify the improvements in head posture in five children with CP after ten work sessions with serious videogames controlled with a head mouse and movements of their heads. This novel therapy was followed in parallel with traditional rehabilitation therapies. We recruited a control group which followed the traditional therapies only, in order to compare the improvements in both groups.

There were improvements in four metrics (VAM, GAS, TCMS, and GMFM-88) in the experimental group, although they were not significant in the case of GMFM-88. The improvements were generally larger for the experimental group and significant differences were found in TCMS between the groups. The percentage of improvement in trunk control is indeed remarkable and shows the potential of this kind biofeedback in rehabilitation therapies.

Despite the lack of statistical significance in the improvements, the values active and passive ROM for flexion, lateral-flexion and extension after ten work sessions are closer to the physiologically normal ROMs, i.e. 55°, 90° and 120° for flexion-extension, inclination, and rotation.

We found some limitations during our experiments which could have influenced our measurements and should be addressed in future trials. To begin with, the motor disorder was more severe in the control group (although not significantly). In addition, a sample size of ten participants (including five controls) could be considered to be too small to provide statistical strength, given the great heterogeneity of the motor disorders of our participants. In addition, the randomization used group the participants led to two groups quite different. However, no significant difference was detected in terms of disability level, and that in age was potentially in favor of the control group. Last, we must bear in mind the fact that we should update the videogames periodically in order to keep our patients motivated.

There are some technical aspects, which also influenced the measurement of the CROM. For instance, the accuracy of the IMU was high enough to enable a comfortable control of the videogames. However, it was far from the one that could have been reached with a motion capture system based on optical markers. Similarly, using only one IMU to estimate the CROM was problematic because any movement in the trunk reflected in the orientation of the head and affected the measurement. The therapists approached this issue by providing the participants with the poorest trunk postural control with pelvic and torso support. Nonetheless, two IMUs located in the head (C0) and torso (T4) are required for a better measurement of the CROM. With this approach CROM could be estimated with the rotation of the IMU located in C0 with respect to the one located in T4.

Unfortunately, we did not performed any analysis of the usability in this study. Nevertheless, the results of previous experiments show that ENLAZA meets some of the criteria stated by ISO 9241-Part 9. “Requirements for non-keyboard input devices”, such as the adherence to Fitts’s law [24]. In addition to this, all the children enrolled in the experimental group, even those of very young age, performed all the planned sessions of rehabilitation. This confirmed the compliance of children in using this device, suggesting its usability in CP. Furthermore, despite it was out of our purposes and due only to randomization process, testing the device on very young children suggest its usability also in older children. Further studies should assess the satisfaction of children, parents and therapists in using this device.

In future experiments, we will use two IMUs to improve the measurement of the CROM. We also intend to recruit a larger group in a multi-center study in order to look for greater significance in the functional and kinematic evaluation. Moreover, we are currently working on the development of a rehabilitation platform with our videogames and other games controlled by movements of the trunk or the upper limbs. Our results suggest that this kind of platform could be a powerful tool for the rehabilitation of CP.

## Conclusion

In this work, we proved that a therapy for the rehabilitation of head and trunk motor control with inertial sensors and serious games as a complement to traditional therapies is possible. Furthermore, we found that the improvements due to this novel therapy are larger than those achieved with traditional therapies alone. Despite our modest sample, the experiments yielded very promising results. Future rehabilitation treatments in physical and occupational therapy will promote the motivation of the patients and be more effective by including the use of adapted serious videogames.

## References

[1] Bax M, Goldstein M, Rosenbaum P, Leviton A, Paneth N, Dan B, Jacobsson B, Damiano D (2005). Proposed definition and classification of cerebral palsy, April 2005. Dev Med Child Neurol.

[2] Blair E (2010). Epidemiology of the cerebral palsies. Orthop Clin N Am.

[3] Johnson A, Perinatal N, Unit E, Road O (2002). Prevalence and characteristics of children with cerebral palsy in Europe. Dev Med Child Neurol.

[4] Cans C (2007). Surveillance of cerebral palsy in Europe: a collaboration of cerebral palsy surveys and registers. Dev Med Child Neurol.

[5] Krägeloh-Mann I, Cans C (2009). Cerebral palsy update. Brain Dev.

[6] Redstone F, West JF (2004). The importance of postural control for feeding. Pediatr Nurs.

[7] Palisano RR, Rosenbaum P, Bartlett D, Livingstone M (2008). Gross motor function classification system—expanded and revised. Child A Glob J Child Res..

[8] Eliasson A-C, Krumlinde-Sundholm L, Rösblad B, Beckung E, Arner M, Ohrvall A-M, Rosenbaum P (2006). The Manual Ability Classification System (MACS) for children with cerebral palsy: scale development and evidence of validity and reliability. Dev Med Child Neurol.

[9] Van der Heide J, Paolicelli PB, Boldrini A, Cioni G (1999). Kinematic and qualitative analysis of lower-extremity movements in preterm infants with brain lesions. Phys Ther.

[10] Kim M, Kim BH, Jo S (2015). Quantitative evaluation of a low-cost noninvasive hybrid interface based on EEG and eye movement. IEEE Trans Neural Syst Rehabil Eng.

[11] Cutti AG, Cappello A, Davalli A (2005). A new technique for compensating the soft tissue artefact at the upper-arm: in vitro validation. J Mech Med Biol.

[12] Ghasemzadeh H, Jafari R, Prabhakaran B (2010). A body sensor network with electromyogram and inertial sensors: multimodal interpretation of muscular activities. IEEE Trans Inf Technol Biomed.

[13] Velasco MA, Raya R, Ceres R, Clemotte A, Ruiz Bedia A, Gonzalez Franco T, Rocon E (2016). Positive and negative motor signs of head motion in cerebral palsy: assessment of impairment and task performance. IEEE Syst J.

[14] Bower E. The multiply handicapped child. In: Wilson BA, et al., McLellan LD, Editors. Rehabilitation studies handbook. New York: Cambridge University Press; 1997. p. 315–54.

[15] Ketelaar M, Vermeer A, Hart H, van Petegem-van Beek E, Helders PJ (2001). Effects of a functional therapy program on motor abilities of children with cerebral palsy. Phys Ther.

[16] Aisen ML, Kerkovich D, Mast J, Mulroy S, Wren TAL, Kay RM, Rethlefsen SA (2011). Cerebral palsy: clinical care and neurological rehabilitation. Lancet Neurol.

[17] Heyrman L, Molenaers G, Desloovere K, Verheyden G, De Cat J, Monbaliu E, Feys H (2011). A clinical tool to measure trunk control in children with cerebral palsy: The Trunk Control Measurement Scale. Res Dev Disabil.

[18] Ledebt A, Becher J, Kapper J, Rozendaalr RM, Bakker R, Leenders IC, Savelsbergh GJP (2005). Balance training with visual feedback in children with hemiplegic cerebral palsy: effect on stance and gait. Mot Control.

[19] Jaume-I-Capó A, Martinez-Bueso P, Moya-Alcover B, Varona J (2014). Interactive rehabilitation system for improvement of balance therapies in people with cerebral palsy. IEEE Trans Neural Syst Rehabil Eng.

[20] Kramer JF, Ashton B, Brander R (1992). Training of head control in the sitting and semi-prone positions. Child Care Health Dev.

[21] Lancioni GE, Singh NN, O’Reilly MF, Sigafoos J, Didden R, Oliva D, Severini L (2007). Fostering adaptive responses and head control in students with multiple disabilities through a microswitch-based program: follow-up assessment and program revision. Res Dev Disabil.

[22] Shih CH, Shih CT, Chu CL (2010). Assisting people with multiple disabilities actively correct abnormal standing posture with a Nintendo Wii Balance Board through controlling environmental stimulation. Res Dev Disabil.

[23] Raya R, Roa JO, Rocon E, Ceres R, Pons JL (2010). Wearable inertial mouse for children with physical and cognitive impairments. Sens Actuators A Phys.

[24] Raya R, Rocon E, Gallego JA, Ceres R, Pons JL (2012). A robust Kalman algorithm to facilitate human-computer interaction for people with cerebral palsy, using a new interface based on inertial sensors. Sensors.

[25] Burke JW, McNeill MDJ, Charles DK, Morrow PJ, Crosbie JH, McDonough SM (2009). Optimising engagement for stroke rehabilitation using serious games. Vis Comput.

[26] Weis R (2004). Gross motor function measure (GMFM-66 and GMFM-88) user’s manual. Eur J Paediatric Neurol..

[27] MacKenzie IS (1989). A note on the information-theoretic basis of Fitts’ law. J Mot Behav.

